# Phase-Specific Nutritional Therapy in Critical Illness: When Protein May Help and When It May Harm, from Acute Metabolic Adaptation to Post-ICU Recovery

**DOI:** 10.3390/nu18172840

**Published:** 2026-08-29

**Authors:** Mircea Stoian, Adina Stoian, Claudia Bănescu, Sergio Rares Bandilă Bandilă, Alina Danilesco, Hajnal Finta, Leonard Azamfirei

**Affiliations:** 1 Department of Anesthesiology and Intensive Care Medicine, George Emil Palade University of Medicine, Pharmacy, Science and Technology of Târgu Mureș, 540103 Târgu Mureș, Romania; mircea.stoian@umfst.ro (M.S.); leonard.azamfirei@umfst.ro (L.A.); 2 Department of Pathophysiology, George Emil Palade University of Medicine, Pharmacy, Science and Technology of Târgu Mureș, 540136 Târgu Mureș, Romania; 3 Department of Genetics, George Emil Palade University of Medicine, Pharmacy, Science and Technology of Târgu Mureș, 540142 Târgu Mureș, Romania; claudia.banescu@umfst.ro; 4 Orthopedic Surgery and Traumatology Service, Marina Baixa Hospital, Av. Alcade En Jaume Botella Mayor, 03570 Villajoyosa, Spain; sergiob1976@gmail.com; 5 Doctoral School of Medicine and Pharmacy, George Emil Palade University of Medicine, Pharmacy, Science and Technology of Târgu Mureș, 540142 Târgu Mureș, Romania; alinka942@yahoo.com; 6 Department of Public Health and Health Management, George Emil Palade University of Medicine, Pharmacy, Science and Technology of Târgu Mureș, 540139 Târgu Mureș, Romania; hajnal.fina@umfst.ro

**Keywords:** critical care nutrition, ICU protein provision, phase-adapted nutrition, anabolic resistance, critical illness, post-intensive care syndrome

## Abstract

**Background:** Nutritional support in critically ill patients remains one of the most debated aspects of intensive care medicine. Although international guidelines have long recommended early nutritional support with progressive protein delivery, recent clinical evidence has questioned the benefit of aggressive early calorie and protein provision during the acute phase of critical illness, highlighting the need for phase-adapted nutritional strategies. **Methods:** This narrative review summarizes current evidence on individualized, phase-adapted nutritional therapy in critically ill adults and post-ICU survivors, focusing on protein dose and timing, anabolic resistance, muscle wasting, and nutritional management during recovery. Relevant studies published between January 2011 and June 2026 were identified through structured searches of PubMed/MEDLINE, supplemented by reference screening and forward citation tracking. A total of 59 publications were included in the final narrative synthesis. **Results:** Recent clinical trials indicate that early high-dose protein administration does not consistently improve survival or functional outcomes and may be associated with harm in selected patient subgroups, particularly those with acute kidney injury, severe organ dysfunction, or shock. More conservative early calorie and protein delivery during the early acute phase has been associated with improved gastrointestinal tolerance and faster clinical stabilization without increasing mortality. Complementary mechanistic evidence supports these findings, highlighting persistent endogenous energy production, anabolic resistance, impaired protein utilization, and reduced autophagy during early critical illness. In contrast, the post-ICU recovery phase is characterized by ongoing muscle wasting, weakness, reduced oral intake, and persistent nutritional deficits, while structured nutritional support after ICU discharge remains inconsistently implemented. **Conclusions:** Current evidence increasingly supports an individualized, phase-adapted approach to nutritional therapy rather than uniform early aggressive calorie and protein targets. Nutritional strategies should consider illness severity, metabolic phase, organ dysfunction, gastrointestinal tolerance, and recovery trajectory. Further studies are needed to define optimal nutritional targets during post-ICU recovery. An evidence-informed practical framework for phase-adapted nutritional therapy is proposed.

## 1. Introduction

How much should we feed a critically ill patient? The question appears deceptively simple. Yet despite decades of research, thousands of patients enrolled in randomized controlled trials, and successive updates of international guidelines, the optimal nutritional strategy in critical illness remains uncertain. Increasing evidence suggests that nutritional requirements depend not only on the amount of energy and protein delivered but also on the timing of nutritional support in relation to the evolving metabolic phases of critical illness [1].

Malnutrition affects 38–78% of critically ill patients, depending on the diagnostic criteria, patient population, and timing of assessment, and is independently associated with increased mortality, prolonged ICU and hospital stay, higher rates of nosocomial infections, and ICU readmission [2,3]. Skeletal muscle wasting begins within hours of ICU admission and progresses rapidly during the first week as a consequence of accelerated proteolysis and impaired protein synthesis, contributing to intensive care unit-acquired weakness (ICU-AW), delayed recovery, and long-term functional disability [4,5]. These observations have traditionally supported early nutritional intervention aimed at attenuating muscle catabolism and preserving lean body mass. Accordingly, current recommendations from the European Society for Clinical Nutrition and Metabolism (ESPEN) and the American Society for Parenteral and Enteral Nutrition (ASPEN) advocate early enteral nutrition together with progressive achievement of energy and protein targets during the acute phase of critical illness [6,7].

Over the past decade, however, this paradigm has been increasingly challenged. A series of landmark randomized controlled trials has questioned whether early achievement of conventional calorie and protein targets improves clinically meaningful outcomes, suggesting instead that aggressive nutritional replacement during the acute phase may be ineffective or even harmful in selected patient populations [8,9,10,11,12,13]. Collectively, these studies indicate that the relationship between nutritional support and outcome is more complex than previously assumed and cannot be adequately addressed using a single nutritional strategy throughout the course of critical illness.

This evolving evidence is biologically plausible. Unlike simple starvation, critical illness induces a profound neuroendocrine and inflammatory response characterized by persistent endogenous glucose production, lipolysis, and proteolysis that continue despite exogenous nutrient delivery [9,14,15]. Simultaneously, anabolic resistance limits the capacity of skeletal muscle to utilize administered amino acids for protein synthesis, while excessive nutrient provision may suppress autophagy, an essential cellular process responsible for maintaining cellular homeostasis during acute stress [3,9,14]. These physiological mechanisms provide a biological rationale for adapting nutritional therapy to the evolving metabolic phases of critical illness, a concept explored in greater detail in the following sections [3,10].

Although increasing attention has been focused on nutritional management during ICU admission, considerably less emphasis has been placed on the recovery period following ICU discharge. Many survivors experience persistent muscle weakness, impaired physical function, reduced oral intake, and the physical component of post-intensive care syndrome (PICS), yet structured nutritional assessment and follow-up remain inconsistently implemented [16,17,18]. This transition from intensive care to hospital wards and community-based rehabilitation therefore represents a critical, but frequently overlooked, component of nutritional care.

A recently published structured narrative review proposed a phase-specific precision-nutrition framework extending from acute feeding to post-ICU recovery. While sharing the concept that nutritional therapy should evolve across the course of critical illness, the present review places particular emphasis on the timing-dependent effects of protein provision and on the clinical circumstances in which higher protein intake may be beneficial, ineffective, or potentially harmful [19].

Against this background, this narrative review summarizes current evidence supporting a phase-adapted approach to nutritional therapy across the continuum of critical illness. The present review integrates phase-specific metabolic physiology, contemporary randomized evidence on protein provision, and post-ICU nutritional recovery into a unified evidence-informed clinical framework.

We discuss the physiological basis of phase-dependent metabolic alterations, critically appraise the evidence from landmark clinical trials that have challenged traditional nutritional paradigms, examine the nutritional needs of post-ICU survivors, and propose a practical framework for individualized nutritional management from ICU admission through recovery.

Building on previous work in personalized nutrition in critically ill patients [20], we propose that nutritional therapy should be individualized not only according to patient characteristics but also according to the metabolic phase of critical illness, thereby integrating precision nutrition with the dynamic pathophysiology of critical illness.

## 2. Materials and Methods

### 2.1. Review Design

This narrative review synthesizes current evidence on phase-specific nutritional therapy in critically ill adults and post-ICU survivors. Given the broad scope of the topic, including pathophysiological mechanisms, clinical trials, international guidelines, and nutritional recovery after intensive care, a narrative approach was considered more appropriate than a systematic review or meta-analysis. The review was prepared in accordance with the Scale for the Assessment of Narrative Review Articles (SANRA) to enhance methodological transparency and reporting quality [21].

The review question was structured according to the PICO framework as follows: the population comprised critically ill adults during ICU admission and post-ICU survivors (P); the interventions or exposures of interest were phase-adapted nutritional strategies involving different timings and doses of energy and protein provision (I); the comparators included conventional early achievement of nutritional targets and alternative nutritional strategies (C); and the outcomes included mortality, clinical outcomes, gastrointestinal tolerance, muscle preservation, and functional recovery (O).

Accordingly, the review question was: In critically ill adults and post-ICU survivors, how do phase-adapted nutritional strategies involving different timings and doses of energy and protein provision, compared with conventional or alternative nutritional strategies, affect mortality, clinical outcomes, muscle preservation, and functional recovery?

### 2.2. Literature Search

A structured PubMed/MEDLINE search was conducted on 20 July 2026 for English-language publications involving critically ill adults and published between 1 January 2011 and 30 June 2026. The Boolean search strategy was: (“critical illness” OR “critically ill” OR “intensive care” OR ICU) AND (nutrition* OR feeding) AND ((protein AND (dose OR timing OR delivery OR provision)) OR “phase-specific” OR “metabolic phase” OR “anabolic resistance” OR “post-ICU” OR “ICU survivor” OR “muscle wasting”). Additional relevant publications were identified through backward reference screening, forward citation tracking, and targeted searches for guidelines and consensus statements.

### 2.3. Eligibility Criteria and Evidence Selection

Eligible publications included randomized and non-randomized clinical trials, systematic reviews, meta-analyses, clinical practice guidelines, and clinically relevant mechanistic or observational studies addressing nutritional therapy in critically ill adults or during post-ICU recovery. Particular emphasis was placed on studies evaluating the timing and dose of energy or protein provision, metabolic adaptation, anabolic resistance, skeletal muscle loss, functional recovery, and the integration of nutrition with rehabilitation.

Studies conducted exclusively in pediatric or neonatal populations, non-peer-reviewed publications, conference abstracts without sufficient methodological information, and articles not directly relevant to nutritional management during or after adult critical illness were excluded. Evidence was selected according to methodological quality, clinical relevance, and contribution to the objectives of the review, with priority given to landmark randomized controlled trials, contemporary meta-analyses, and international guidelines.

Titles and abstracts of 429 PubMed records were screened for relevance, followed by full-text assessment of 86 publications. After full-text assessment, 21 publications were selected from the PubMed search. An additional 37 relevant publications were identified and included through citation tracking and targeted searches for guidelines and consensus statements. One additional relevant publication was identified and included during peer-review revision, resulting in a total of 59 publications in the revised narrative synthesis.

The literature identification and evidence-selection process is presented in a PRISMA-style flow diagram adapted to the narrative-review design (Supplementary Figure S1).

### 2.4. Methodological Considerations

As a narrative review, this study does not include a formal risk-of-bias assessment or quantitative evidence synthesis. To enhance the robustness of the synthesis and reduce selection bias, predefined eligibility criteria were applied, the structured database search was supplemented by backward reference screening and forward citation tracking, and the evidence-selection process was transparently documented. Priority was given to high-quality randomized trials, contemporary meta-analyses, and international clinical practice guidelines, while areas of conflicting or limited evidence were explicitly acknowledged throughout the review [21].

Nevertheless, residual selection bias cannot be excluded because study selection was not conducted as a formal systematic-review process.

Given the narrative design and the heterogeneity of the evidence considered, study identification and selection were intended to provide a clinically representative synthesis rather than an exhaustive systematic retrieval of all available publications.

## 3. Metabolic Basis of Phase-Specific Nutritional Therapy

### 3.1. Metabolic Response to Critical Illness

Critical illness elicits a complex neuroendocrine and inflammatory response that differs fundamentally from the metabolic adaptations observed during simple starvation. Rather than conserving energy stores, the organism enters a hypercatabolic state characterized by accelerated glycogenolysis, gluconeogenesis, lipolysis, and proteolysis, driven by increased concentrations of cortisol, catecholamines, glucagon, and pro-inflammatory cytokines [1,2]. Recent physiological models describe this response as a dynamic process that evolves through distinct metabolic phases, each characterized by specific endocrine, inflammatory, gastrointestinal, and mitochondrial adaptations with important implications for nutritional therapy [1,3].

A hallmark of the early phase of critical illness is persistent endogenous substrate production. Unlike physiological fasting, endogenous glucose and lipid mobilization are only minimally suppressed by exogenous nutrient delivery, meaning that administered calories and protein are added to, rather than replacing, ongoing endogenous substrate production [1,5]. Consequently, early achievement of calculated energy targets may increase the risk of overfeeding without fully attenuating the underlying catabolic response [1,4].

Instead, excessive protein delivery may increase nitrogen and urea production while imposing an additional metabolic burden, particularly in patients with impaired renal or hepatic function. These physiological adaptations provide a mechanistic rationale for the phase-adapted nutritional strategies discussed in subsequent sections [10,11,22].

### 3.2. Cellular Adaptation: Autophagy and Mitochondrial Function

Beyond systemic metabolic alterations, critical illness is accompanied by profound intracellular adaptations that influence the response to nutritional therapy. Among these, autophagy plays a central role in maintaining cellular homeostasis by removing damaged organelles, dysfunctional mitochondria, and misfolded proteins, thereby facilitating cellular repair during acute stress [23,24]. Experimental and human studies suggest that insufficient activation of autophagy may contribute to the accumulation of cellular damage and persistent organ dysfunction during critical illness [8,23].

The interaction between nutritional support and autophagy has attracted increasing attention over the past decade. Experimental evidence indicates that early nutrient administration, particularly in excess of metabolic requirements, may attenuate autophagic activity, whereas a transient macronutrient deficit may preserve this adaptive cellular response [14,25]. Consistent with this concept, a predefined analysis of the EPaNIC trial reported a lower incidence of ICU-acquired weakness in patients exposed to a greater early macronutrient deficit, providing indirect clinical support for the hypothesis that permissive underfeeding during the acute phase may favor endogenous cellular repair mechanisms [9]. Although a direct causal relationship has not been established, these observations offer a biologically plausible explanation for the neutral or unfavorable outcomes reported in several trials evaluating aggressive early nutritional support [6,8,9].

Mitochondrial function represents another important component of the metabolic response to critical illness. Rather than reflecting a simple failure of energy production, current evidence supports the concept of adaptive metabolic downregulation, characterized by reduced oxidative phosphorylation and greater reliance on alternative energy substrates, including glycolysis, lactate, and, under selected conditions, ketone bodies [26]. This adaptive response may protect cells during the acute phase of illness but also limits the capacity to efficiently utilize exogenous nutrients.

Together with anabolic resistance and altered autophagy, these mechanisms provide a coherent physiological rationale for avoiding excessive calorie and protein delivery during the early phase of critical illness.

### 3.3. The Metabolic Phases of Critical Illness

The physiological mechanisms described above support the concept that critical illness is a dynamic process rather than a single metabolic state. Current physiological models describe three broad metabolic phases with distinct nutritional implications: an early acute phase characterized by persistent endogenous substrate production, anabolic resistance, and adaptive cellular responses; a stabilization phase during which the inflammatory and neuroendocrine stress response gradually subsides; and a recovery phase, in which anabolic responsiveness progressively returns and nutritional requirements increase to support tissue repair, muscle protein synthesis, and functional rehabilitation [1,3,20].

This evolving metabolic trajectory suggests that nutritional therapy should be adapted to the patient’s phase of illness rather than guided by uniform calorie and protein targets throughout the entire ICU stay. During the acute phase, conservative nutritional strategies may better accommodate the underlying metabolic response, whereas delayed recovery or insufficient nutritional support during the anabolic phase may contribute to persistent muscle wasting, ICU-acquired weakness, and poor functional recovery [1,2,20].

Despite growing recognition of this phase-specific model, translating it into routine clinical practice remains challenging. No validated bedside biomarker currently identifies the transition from the acute catabolic phase to anabolic recovery, and clinicians must rely on the integration of clinical evolution, organ function, inflammatory status, and nutritional tolerance when individualizing nutritional therapy.

**Figure 1:**
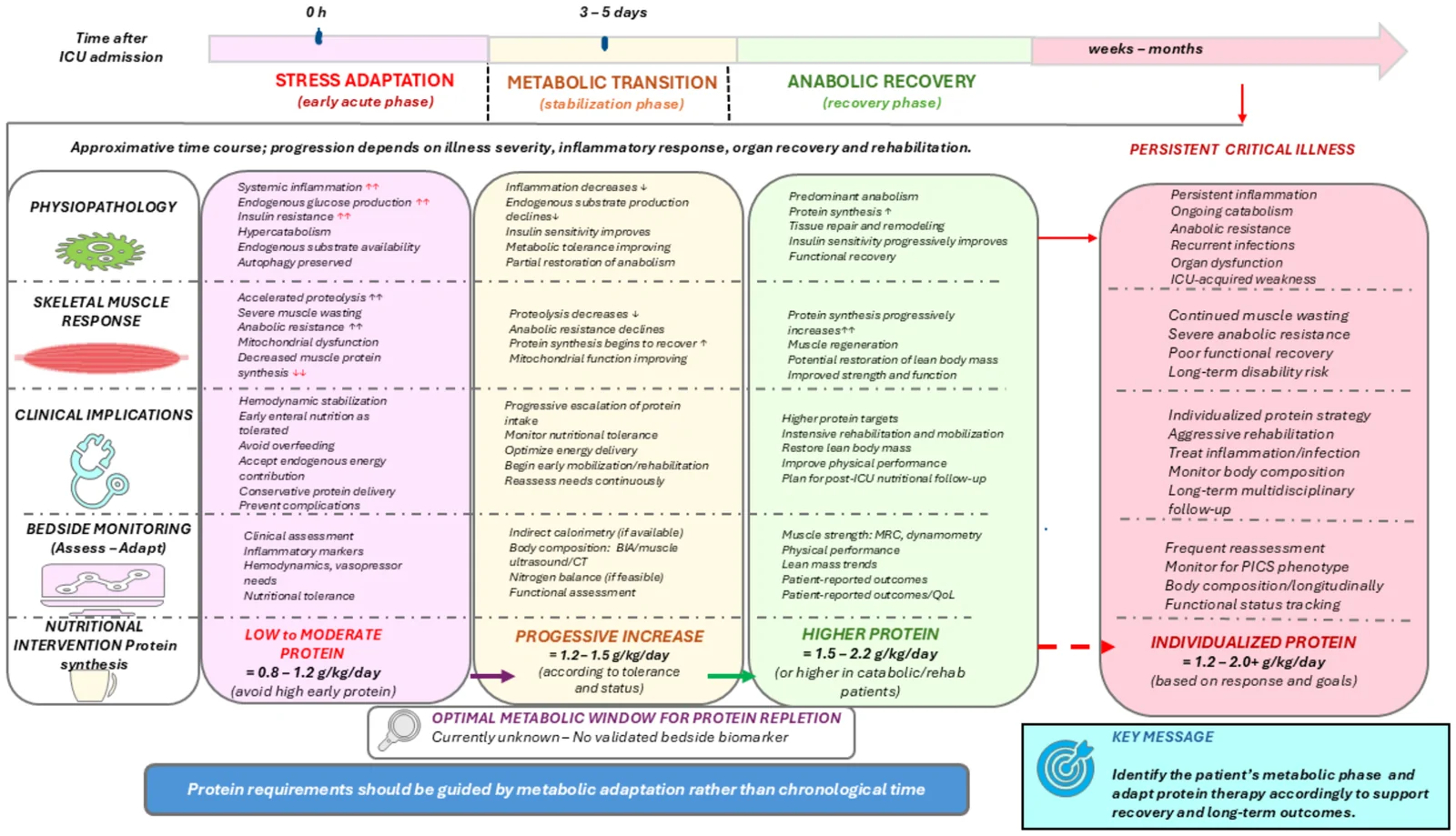
Dynamic metabolic evolution during critical illness: translating pathophysiology into phase-adapted nutritional therapy. The figure is original and was created by the authors specifically for this review. Notes: The figure provides a conceptual representation of the physiological basis of phase-adapted nutritional therapy. It summarizes the dynamic metabolic continuum of critical illness, the principal mechanisms characterizing each phase, their consequences for skeletal muscle, and the clinical indicators that may assist in assessing metabolic progression. Progression between phases is driven primarily by physiological recovery rather than chronological time. Because no validated bedside biomarker currently identifies the transition from catabolism to anabolic recovery, the indicators shown should be interpreted collectively and not as validated phase-defining criteria. The transitions between phases are gradual and should not be interpreted as discrete clinical states. The physiological concepts illustrated here provide the rationale for the practical framework presented later in this review. Abbreviations: Abbreviations: BIA, bioelectrical impedance analysis; CT, computed tomography; ICU, intensive care unit; MRC, Medical Research Council; PICS, post-intensive care syndrome; QoL, quality of life.

The metabolic response to critical illness represents a dynamic continuum rather than a series of fixed chronological stages. Figure 1 provides a conceptual representation of this continuum by integrating the principal pathophysiological mechanisms, their effects on skeletal muscle, and the corresponding nutritional implications. Defining reliable markers of metabolic transition remains an important research priority and is discussed in subsequent sections of this review.

## 4. Nutritional Therapy During the Acute Phase

As illustrated in Figure 1, the metabolic response to critical illness evolves through distinct but overlapping phases, providing the physiological rationale for a phase-adapted approach to protein administration.

### 4.1. From Observational Studies to Randomized Clinical Trials

Early recommendations supporting aggressive protein administration were largely based on observational studies showing an association between higher protein delivery and improved clinical outcomes [27,28]. However, these studies are inherently susceptible to confounding by indication, as patients with less severe illness generally tolerate enteral feeding more successfully and therefore receive greater amounts of nutrition. Consequently, higher protein intake may reflect a patient’s clinical stability rather than represent the direct cause of improved outcomes [29,30].

The PROTINVENT study illustrates this limitation. Early high protein administration was associated with lower mortality among non-septic mechanically ventilated patients, whereas delayed achievement of higher protein intake was associated with better outcomes in the overall study population [30]. These observations highlight that protein timing, patient selection, and tolerance to nutritional therapy may all influence observed associations, emphasizing the need for randomized controlled trials capable of minimizing residual confounding.

### 4.2. Evidence from Landmark Randomized Controlled Trials

The paradigm established by observational studies has been substantially challenged by recent randomized controlled trials. The EPaNIC trial first demonstrated that delaying supplemental parenteral nutrition until the second week of critical illness resulted in fewer infections, shorter ICU stay, and faster recovery than early supplementation, suggesting that complete nutritional replacement during the acute phase may not be beneficial [14]. Importantly, EPaNIC primarily evaluated the timing of supplemental parenteral nutrition rather than the isolated effect of protein dose.

Subsequent trials extended these observations. NUTRIREA-3 showed that a restrictive calorie–protein strategy during the first week of critical illness was associated with earlier readiness for ICU discharge and fewer gastrointestinal complications without increasing mortality in mechanically ventilated patients with shock [10]. Thus, NUTRIREA-3 evaluated a combined calorie–protein strategy rather than the independent effect of protein dose. Because both calorie and protein delivery differed between study groups, the independent contribution of protein restriction could not be determined, but the trial clearly challenged the concept of routine early nutritional replacement.

Unlike EPaNIC and NUTRIREA-3, the following trials were specifically designed to evaluate the effect of protein dose while minimizing differences in energy delivery. EFFORT Protein specifically evaluated higher protein administration while allowing usual energy delivery. High-dose protein did not improve time-to-discharge alive or mortality and was associated with an unfavorable signal in patients with acute kidney injury, although these subgroup findings require prospective confirmation [11,31].

PRECISe provided one of the most rigorous assessments of protein dose by comparing two enteral protein targets under double-blind conditions while maintaining similar caloric intake. Higher early protein administration may adversely affect patient-centred outcomes under selected clinical conditions [12]. These findings were subsequently reinforced by a pre-planned Bayesian reanalysis [32].

Similarly, the isocaloric TARGET Protein trial extended these observations to a broad, unselected ICU population. Increasing enteral protein delivery did not improve hospital-free days or 90-day survival, suggesting that the absence of benefit associated with aggressive early protein administration is not limited to highly selected ICU populations [33].

Not all randomized evidence has been neutral or unfavorable. Nakamura et al. reported less femoral muscle-volume loss with higher isocaloric protein delivery, although this benefit was observed primarily when higher protein was combined with active early rehabilitation [34]. More recently, Badpeyma et al. reported lower in-hospital mortality and reduced loss of mid-arm circumference with higher protein provision in a small randomized trial; however, 60-day mortality was not significantly different, and the limited sample size restricts the generalizability of these findings [27]. Collectively, these results suggest that the response to higher protein provision may depend on patient selection, concomitant anabolic stimulation, and the timing of nutritional therapy. Although these trials addressed different nutritional questions—including the timing of nutritional support, combined calorie–protein strategies, and isolated protein-dose interventions—the larger contemporary trials have not demonstrated a clinically meaningful benefit from routine early high-dose protein administration. The favorable signals observed in smaller studies require confirmation and may depend on concomitant rehabilitation, patient selection, and the timing of protein delivery.

### 4.3. Evidence from Meta-Analyses

Recent meta-analyses have confirmed the overall consistency of these randomized data. A systematic review with trial sequential analysis demonstrated no overall mortality benefit from higher protein delivery while identifying a significant increase in mortality among patients with acute kidney injury together with higher serum urea concentrations [29]. A subsequent Bayesian meta-analysis reached similar conclusions, showing little probability that higher early protein administration improves clinically relevant outcomes across the available randomized evidence [28].

Although the included trials differed in patient populations, nutritional protocols, and protein delivery strategies, the overall findings were broadly consistent across randomized trials. Overall, the available randomized evidence does not support routine early high-dose protein administration during the acute phase of critical illness. The characteristics and main findings of the key randomized controlled trials and evidence syntheses discussed in this section are summarized chronologically in Table 1.

### 4.4. Clinical Interpretation

Taken together, the available physiological and clinical evidence supports a phase-specific approach to nutritional therapy during acute critical illness. The metabolic adaptations discussed above provide a biologically plausible explanation for why aggressive early calorie and protein delivery has not consistently translated into improved clinical outcomes in randomized trials [1,3,9,14,26]. Importantly, these findings should not be interpreted as evidence against nutritional support. Rather, they suggest that during the acute phase, the capacity to efficiently utilize exogenous nutrients is limited, and early achievement of full calorie and protein targets may not provide the expected anabolic benefit [10,11,12,13,33,35]. Conversely, a more phase-adapted nutritional strategy appears to be safe in most critically ill patients and may reduce complications in selected populations, although the optimal nutritional targets are likely to vary according to illness severity, organ dysfunction, and individual metabolic status [10,11,12,13,33,35].

Current evidence therefore supports an individualized, phase-specific nutritional strategy rather than the routine application of uniform calorie and protein targets throughout the ICU stay [1,12]. Importantly, these findings should not be interpreted as advocating undernutrition or delaying appropriate nutritional support. Consequently, nutritional management should integrate the patient’s clinical evolution, organ function, nutritional tolerance, and overall recovery trajectory when individualizing nutritional therapy. The nutritional management of this recovery phase is discussed in the following chapter.

## 5. Nutritional Therapy During the Stabilisation and Recovery Phase

### 5.1. Transitioning from Restriction to Repletion

The evidence presented in the previous chapter applies primarily to the acute phase of critical illness, generally encompassing the first 5–7 days of ICU admission, and should not be interpreted as supporting prolonged calorie or protein restriction. As the neuroendocrine stress response gradually resolves, endogenous substrate production declines and anabolic responsiveness progressively recovers, increasing the capacity to utilize exogenous nutrients for tissue repair, muscle protein synthesis, and functional recovery [1,3]. Consequently, nutritional priorities shift from avoiding overfeeding to preventing cumulative energy and protein deficits that may impair rehabilitation.

The principal challenge is that this metabolic transition cannot yet be identified by a validated bedside biomarker. No laboratory parameter, imaging modality, or physiological threshold reliably defines the point at which an individual patient moves from predominant catabolism to anabolic recovery [3]. Consequently, clinicians currently rely on a combination of clinical evolution, improving organ function, resolution of shock and inflammation, nutritional tolerance, and the overall trajectory of recovery when progressively advancing nutritional support.

Although no tool directly identifies the metabolic transition, the modified Nutrition Risk in the Critically Ill (mNUTRIC) score may assist clinical decision-making by identifying patients at greater nutritional risk once the acute phase has resolved. Importantly, mNUTRIC was developed as a risk-stratification instrument rather than a guide for determining calorie or protein targets and should not be used to justify aggressive nutritional support during the early hypercatabolic phase [11].

### 5.2. Progressive Protein Repletion During Recovery

Compared with the acute phase, evidence guiding nutritional therapy during the stabilisation and recovery phases remains considerably more limited. Nevertheless, the available data consistently suggest that progressively increasing protein delivery becomes physiologically appropriate as anabolic responsiveness returns [1,3].

In a randomized trial comparing higher- and moderate-protein administration under similar energy delivery, patients receiving approximately 1.8 g/kg/day of protein after the initial acute phase achieved improved nitrogen balance and reduced skeletal muscle loss without the adverse renal or functional signals reported in trials evaluating high protein administration from ICU admission [34]. Although these findings require confirmation in larger multicentre studies, they support the concept that the effectiveness of protein therapy depends not only on dose but also on timing relative to the patient’s metabolic phase.

Protein supplementation alone, however, is unlikely to fully restore muscle mass or function. Recovery from critical illness requires the integration of adequate nutritional support with early mobilisation and structured rehabilitation. In the IGREEN study, a multimodal strategy combining higher protein delivery, early mobilisation, and neuromuscular electrical stimulation was associated with attenuated loss of quadriceps muscle mass compared with standard care [36]. Similarly, the EMAS randomized trial demonstrated that combining guideline-based nutritional support with early mobilisation reduced the incidence of ICU-acquired weakness compared with mobilisation alone or standard care [37]. Collectively, these findings suggest that the effectiveness of nutritional therapy during recovery depends not only on protein provision but also on its integration with structured rehabilitation.

### 5.3. Clinical Implications

The evidence reviewed throughout this article indicates that nutritional therapy in critically ill patients should be viewed as a dynamic process rather than the pursuit of fixed calorie and protein targets. During the acute phase, physiological adaptations—including persistent endogenous substrate production, anabolic resistance, and altered cellular metabolism—limit the effective utilization of exogenous nutrients, providing a biological rationale for a conservative nutritional strategy [1,3,9,23,26]. As patients stabilize and enter the recovery phase, these adaptations progressively resolve, increasing the potential benefit of higher protein provision, particularly when combined with early mobilization and structured rehabilitation [34,37,38].

Although current evidence supports this phase-specific approach, important knowledge gaps remain. The precise timing of transition from restrictive to anabolic nutritional therapy has not been established, and no validated bedside biomarker currently identifies this metabolic shift [1,3]. Consequently, nutritional management should be individualized by integrating the patient’s clinical course, recovery of organ function, nutritional tolerance, and rehabilitation goals rather than relying solely on predefined calorie or protein targets. This concept forms the basis of the practical phase-specific nutritional algorithm proposed later in this review.

### 5.4. When Recovery Is No Longer the Goal

The phase-specific nutritional approach described above assumes that recovery remains the principal therapeutic objective. However, a proportion of critically ill patients experience progressive irreversible organ failure or transition to end-of-life care, where the goals of nutritional management fundamentally change. In this context, decisions regarding clinically assisted nutrition and hydration (CANH) should be guided primarily by the patient’s goals of care, expected prognosis, symptom burden, and ethical considerations rather than by conventional nutritional targets [38,39].

Reduced nutritional intake during the dying process is frequently a consequence of advanced disease rather than its cause, and escalation of artificial nutrition or hydration does not necessarily improve comfort, quality of life, or survival [33]. Consequently, decisions regarding the continuation, withholding, or withdrawal of CANH require individualized multidisciplinary assessment together with clear communication involving patients, families, and the healthcare team [38].

Differences in the perception of end-of-life nutrition among healthcare professionals, trainees, and the general public further highlight the importance of structured communication and shared decision-making [39]. Therefore, a comprehensive phase-specific nutritional strategy should include regular reassessment of the patient’s overall trajectory of care, recognizing that, when recovery is no longer an achievable goal, nutritional interventions should be aligned with comfort-focused and patient-centred care rather than continued escalation of calorie and protein delivery [38,39].

## 6. Bridging the Post-ICU Nutritional Gap

### 6.1. Nutritional Challenges After ICU Discharge

Although nutritional management during ICU admission has received increasing attention, the period following ICU discharge remains comparatively underexplored. An estimated 84% of ICU survivors continue to require nutritional support after transfer to the hospital ward, while almost all patients leave the ICU with persistent physical impairment and ongoing nutritional vulnerability [16]. As ICU survival continues to improve, the number of patients requiring prolonged nutritional rehabilitation is increasing, making post-ICU nutrition an essential component of the recovery pathway rather than a continuation of acute care [33,40].

Persistent muscle wasting, malnutrition, and inadequate nutritional intake contribute substantially to the physical domain of post-intensive care syndrome (PICS), reinforcing the functional limitations that originate during critical illness [17]. Although adequate protein and energy provision are increasingly recognized as important determinants of recovery after the acute phase, nutritional management following ICU discharge remains inconsistently implemented and is only briefly addressed in current clinical practice guidelines [6]. The recently established Global Research Initiative on Post-ICU Nutrition (GRIP) has highlighted this gap by identifying post-ICU nutritional care as a major international research priority requiring dedicated clinical studies and implementation strategies [41].

### 6.2. Barriers to Nutritional Recovery

The limited implementation of structured post-ICU nutritional care reflects several organizational and clinical barriers. An international survey conducted by the ESPEN Special Interest Group on Nutrition in the ICU demonstrated that formal post-ICU follow-up programmes remain uncommon, and even when available, nutritional assessment is inconsistently incorporated into routine follow-up [18]. Similarly, qualitative studies have identified persistent dysphagia, poor appetite, altered taste, fatigue, and fragmented transitions between hospital and community care as major barriers to achieving adequate nutritional intake after hospital discharge [42].

These observations are consistent with the limited guidance currently provided by international recommendations. Recent evidence suggests that nutritional therapy after ICU discharge should not be considered an isolated intervention but rather an integral component of multidisciplinary rehabilitation. Although high-quality evidence remains limited, combining tailored protein intake with structured physical rehabilitation appears more promising than nutritional supplementation alone for improving functional recovery, muscle strength, and quality of life [18,43]. These findings support the need for patient-specific nutritional strategies integrated with multidisciplinary rehabilitation throughout the post-ICU recovery period.

Despite increasing recognition of the importance of post-ICU nutrition, practical implementation remains inconsistent because of organizational barriers, limited multidisciplinary follow-up, and the scarcity of interventional evidence [6,7,42,43]. This evidence gap has become one of the principal motivations for the GRIP initiative, which identified post-ICU nutritional assessment, personalized nutritional interventions, long-term monitoring, and implementation strategies among its highest research priorities.

### 6.3. Towards Structured Nutritional Continuity

Despite the limited interventional evidence, several practical principles may improve continuity of nutritional care across the recovery pathway:

- Nutritional risk should be reassessed at ICU discharge rather than assuming that nutritional vulnerability resolves with transfer to a general ward.
- An individualized nutrition plan should accompany every transition of care, including transfers from the ICU to the ward and from hospital to community care.
- Routine screening for dysphagia, impaired appetite, and inadequate oral intake should be incorporated into post-ICU assessment to identify potentially reversible barriers to nutritional recovery.
- Nutritional follow-up should be integrated with multidisciplinary post-ICU rehabilitation programmes whenever available, recognizing that nutritional therapy and physical rehabilitation are complementary components of functional recovery [18,41,43].

### 6.4. Clinical Perspective

Current evidence and expert consensus indicate that nutritional management should extend beyond ICU discharge and be considered an integral component of long-term recovery rather than the final step of intensive care. Although high-quality randomized studies evaluating post-ICU nutritional interventions remain limited, available observational evidence, expert consensus, and international initiatives consistently support structured nutritional reassessment, individualized follow-up, and closer integration of nutrition with multidisciplinary rehabilitation [10,33,41]. These principles provide the conceptual bridge between phase-specific nutritional therapy and the individualized management strategies presented in the following section.

## 7. Individualising Phase-Specific Nutritional Therapy

### 7.1. Principles of Individualisation

The phase-specific approach described in the previous sections provides a conceptual framework for nutritional management during critical illness. However, translating this framework into bedside practice requires individualized assessment of nutritional risk, energy expenditure, and the patient’s evolving metabolic status. At present, no single tool can accurately determine both nutritional requirements and metabolic phase; therefore, individualized nutritional therapy relies on integrating physiological measurements, structured nutritional assessment, and clinical judgement [1,3].

### 7.2. Measuring Energy Requirements

Indirect calorimetry remains the reference method for estimating resting energy expenditure and is recommended by current ESPEN guidelines whenever available [6]. By directly measuring oxygen consumption and carbon dioxide production, it provides the most accurate assessment of energy expenditure and allows caloric prescription to be adjusted throughout the course of critical illness.

Despite these advantages, indirect calorimetry remains unavailable in many intensive care units because of equipment costs, technical requirements, and limited availability of trained personnel. Consequently, predictive equations based on body weight remain the most widely used approach in routine practice [6,7]. However, these equations provide only approximate estimates of energy expenditure and may be particularly inaccurate in patients with obesity, fluid overload, or marked alterations in body composition [44,45,46].

Ventilator-derived carbon dioxide production (VCO_2_) has emerged as a practical alternative for mechanically ventilated patients. By applying a modified Weir equation to routinely measured VCO_2_, clinicians can obtain estimates of energy expenditure that are generally more accurate than predictive equations while avoiding the technical complexity of indirect calorimetry [44]. Although this approach requires further validation across different patient populations and ventilatory settings, it may represent a feasible strategy for improving individualized energy prescription where indirect calorimetry is unavailable.

### 7.3. Nutritional Risk Assessment

Assessment of nutritional risk complements the estimation of energy expenditure by identifying patients most likely to benefit from individualized nutritional interventions. The Nutritional Risk Screening 2002 (NRS-2002) remains a widely used screening tool for identifying patients at risk of malnutrition across hospital settings [47]. For critically ill patients, the NUTRIC and modified NUTRIC (mNUTRIC) scores provide a more specific assessment by identifying individuals with a higher nutritional risk and potentially greater benefit from optimized nutritional support once the acute phase has resolved [11].

The Global Leadership Initiative on Malnutrition (GLIM) criteria have further standardized the diagnosis of malnutrition by combining phenotypic and etiological criteria, thereby facilitating a more consistent assessment across different healthcare settings [48]. Importantly, these instruments identify nutritional risk or established malnutrition but were not designed to determine metabolic phase. Consequently, they should support, rather than replace, individualized clinical decision-making.

### 7.4. Current Limitations of Nutritional Assessment

Despite considerable advances in nutritional assessment, an important gap remains between estimating nutritional requirements and identifying the optimal timing for nutritional intervention. Current tools can estimate energy expenditure, assess nutritional risk, diagnose malnutrition, and monitor changes in body composition, but none reliably identifies the transition from acute catabolism to anabolic recovery. Candidate biomarkers related to autophagy, mitochondrial function, and metabolic regulation remain insufficiently validated for routine clinical use [1,3,49,50].

In current practice, individualized nutritional therapy therefore depends on the integration of indirect calorimetry or VCO_2_-derived estimates of energy expenditure, structured nutritional risk assessment, serial evaluation of body composition when feasible, clinical evidence of organ recovery, nutritional tolerance, and rehabilitation goals [51,52,53,54,55,56,57].

## 8. A Practical Phase-Adapted Framework

### 8.1. Proposed Clinical Framework

**Figure 2:**
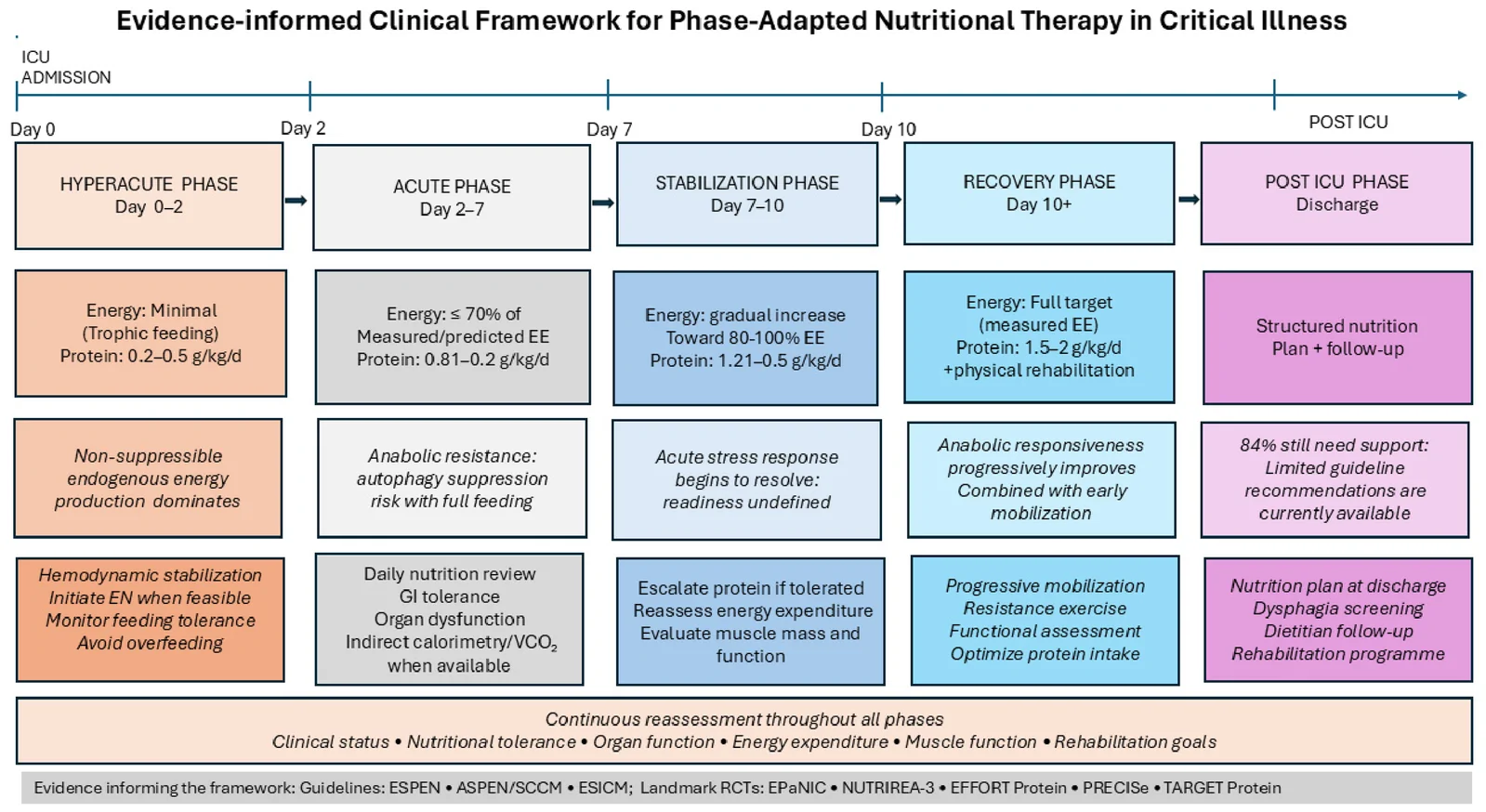
Evidence-informed clinical framework for phase-adapted nutritional therapy across the continuum of critical illness. The figure is original and was created by the authors specifically for this review. Notes: This figure operationalizes the physiological concepts illustrated in Figure 1 by translating them into a practical framework for individualized nutritional management. The algorithm summarizes the progression of calorie and protein delivery from the hyperacute and acute phases through stabilization, recovery, and post-ICU care, while integrating nutritional assessment, metabolic monitoring, body composition evaluation, organ recovery, nutritional tolerance, and rehabilitation. The framework represents an evidence-informed synthesis developed by the authors and has not been prospectively validated as a unified clinical protocol. The suggested time intervals are intended as approximate clinical guidance and should be adapted according to the individual metabolic response and clinical trajectory rather than applied as fixed chronological cut-offs.

Building upon the physiological concepts summarized in Figure 1, Figure 2 translates the phase-specific metabolic model into an evidence-informed clinical framework for individualized nutritional therapy across the continuum of critical illness. Rather than representing a rigid protocol, the proposed framework integrates four complementary dimensions of nutritional management: phase-specific energy and protein targets, the physiological rationale supporting each metabolic phase, key bedside actions required during clinical care, and continuous reassessment throughout the patient’s trajectory.

The framework further synthesizes evidence from contemporary international guidelines together with landmark randomized clinical trials, emphasizing that nutritional therapy should evolve according to metabolic adaptation rather than predefined chronological milestones. Its purpose is not to prescribe fixed nutritional targets but to facilitate individualized clinical decision-making by integrating the patient’s metabolic phase, organ recovery, nutritional tolerance, functional status, and rehabilitation goals. Accordingly, the proposed framework should be regarded as an evidence-informed clinical decision-support tool rather than a validated clinical algorithm.

The proposed framework highlights several principles that may facilitate bedside implementation. First, nutritional therapy should evolve according to the patient’s metabolic trajectory rather than follow uniform calorie and protein targets throughout the ICU stay. During the hyperacute and acute phases, conservative calorie and protein administration appears appropriate while endogenous substrate production remains elevated and anabolic resistance limits the effective utilization of exogenous nutrients [1,3,11,12,13,23,26,33,35,50]. At the bedside, this strategy requires continuous assessment of hemodynamic stability, gastrointestinal tolerance, and organ function before nutritional support is progressively advanced. As patients stabilize and enter the recovery phase, nutritional priorities progressively shift toward preventing cumulative protein deficits, preserving lean body mass, and supporting functional recovery through increased protein provision combined with early mobilization and rehabilitation [1,3,34,36,37].

Second, the transition between metabolic phases should not be regarded as a fixed chronological event. Although the approximate time intervals illustrated in the framework reflect the intervention periods used in landmark randomized clinical trials, they should be interpreted as practical clinical guidance rather than validated cut-off values. Because no validated bedside biomarker currently identifies the transition from acute catabolism to anabolic recovery, progression between phases should be guided by continuous reassessment integrating clinical evolution, recovery of organ function, nutritional tolerance, measured or estimated energy expenditure, muscle mass and function, and rehabilitation goals rather than ICU length of stay alone [1,3].

Third, the framework explicitly extends nutritional care beyond ICU discharge. Structured nutrition plans, dysphagia screening, assessment of appetite and oral intake, dietitian follow-up, and integration with multidisciplinary rehabilitation are presented as essential components of nutritional continuity despite the limited guidance currently provided by international recommendations. By incorporating the post-ICU period into the same clinical framework, the framework emphasizes that nutritional therapy should be viewed as a continuous process extending from ICU admission through long-term functional recovery [12,38,39].

### 8.2. Future Research Directions

Despite substantial advances in critical care nutrition, several important knowledge gaps remain. The highest priority is the identification and validation of reliable bedside biomarkers capable of defining the transition from acute catabolism to anabolic recovery, thereby allowing nutritional therapy to be individualized according to metabolic phase rather than chronological time. Candidate biomarkers related to metabolic regulation, mitochondrial function, and autophagy remain investigational and require prospective clinical validation [1,3].

Additional randomized controlled trials are needed to determine the optimal protein targets during the stabilization and recovery phases of critical illness and to clarify their interaction with early mobilization and structured rehabilitation programmes [34,36,37]. Equally important is the development of interventional studies evaluating structured nutritional follow-up after ICU discharge, an area that remains poorly represented in current evidence despite its increasing clinical importance [10,41].

Several ongoing randomized trials are expected to address these knowledge gaps. The multicentre REPLENISH trial is evaluating a stepwise increase in enteral protein provision commencing on ICU day 5, with 90-day all-cause mortality as the primary outcome [58]. By delaying protein escalation beyond the earliest phase of critical illness, this trial may help clarify whether the timing of protein repletion influences clinical outcomes. A separate randomized trial is evaluating higher-protein enteral nutrition combined with standardized advanced early mobilization, directly addressing the proposed interaction between protein provision and rehabilitation [59].

Finally, future studies should evaluate nutritional strategies in specific high-risk populations, including patients with acute kidney injury, obesity, frailty, and pre-existing sarcopenia, who may require more individualized nutritional approaches than those currently recommended for the general ICU population.

### 8.3. Strengths and Limitations of This Review

This review integrates recent physiological concepts, randomized clinical trials, post-ICU nutritional care, and individualized nutritional assessment into a unified phase-specific framework for nutritional therapy in critically ill adults. By combining mechanistic evidence with practical clinical recommendations, it provides a comprehensive overview of nutritional management across the entire continuum of critical illness, from ICU admission to post-discharge recovery.

Nevertheless, several limitations should be acknowledged. First, as a narrative review, no formal risk-of-bias assessment or GRADE evaluation was performed, and residual selection bias cannot be excluded despite the structured search and predefined eligibility criteria. Second, the literature search was restricted to publications in English, potentially excluding relevant studies published in other languages. Third, the evidence synthesized in this review remains heterogeneous with respect to patient populations, nutritional interventions, outcome measures, and study designs. Finally, the proposed phase-adapted framework represents an evidence-informed synthesis rather than a prospectively validated clinical protocol and should therefore be interpreted as a practical approach requiring further prospective evaluation before widespread implementation.

## 9. Conclusions

Recent physiological and clinical evidence has challenged the traditional concept that early high-calorie and high-protein delivery uniformly benefits critically ill patients. Instead, nutritional therapy should be adapted to the patient’s metabolic phase, recognizing that nutritional requirements evolve throughout the course of critical illness.

Current evidence supports a phase-adapted nutritional strategy, with conservative calorie and protein delivery during the acute phase followed by progressive protein repletion during stabilization and recovery, ideally combined with early rehabilitation. Equally important, nutritional care should extend beyond ICU discharge, where persistent malnutrition and muscle loss continue to compromise functional recovery despite limited guidance from current recommendations.

Rather than pursuing higher protein delivery alone, individualized nutritional therapy should integrate metabolic monitoring, body composition assessment, and structured rehabilitation. Together, the physiological concepts and evidence-informed clinical framework proposed in this review provide a practical foundation for phase-adapted precision nutrition across the continuum of critical illness.

## Figures and Tables

**Table 1:** Summary of key randomized controlled trials and evidence syntheses evaluating nutritional strategies during acute critical illness.

Study/Year	*n*/Design	Key Evidence	Clinical Implication
**Casaer et al.** **EPaNIC** 2011 [14]	*n* = 4640; multicentre RCT; early versus late supplemental PN	PN initiated on days 1–2 increased infections and prolonged ICU stay and recovery compared with initiation on day 8, without reducing mortality.	Early completion of nutritional targets during acute critical illness may not improve outcomes and may cause harm.
**Nakamura et al., 2021** [34]	*n* = 117; RCT; isocaloric high- versus medium-protein delivery	Achieved protein intake was approximately 1.5 versus 0.8 g/kg/day. Higher protein reduced femoral muscle-volume loss primarily when combined with active rehabilitation.	The effect of protein provision may depend on concomitant rehabilitation and anabolic stimulation.
**Reignier et al.** **NUTRIREA-3** 2023 [10]	*n* = 3044; multicentre RCT; ventilated adults with shock in 61 French ICUs	Low targets of 6 kcal/kg/day and 0.2–0.4 g protein/kg/day versus 25 kcal/kg/day and 1.0–1.3 g/kg/day during days 1–7. Restriction accelerated readiness for ICU discharge and reduced gastrointestinal and hepatic complications without affecting 90-day mortality.	Supports conservative calorie and protein delivery during the early acute phase in ventilated patients with shock.
**Heyland et al.** **EFFORT Protein** **2023** [11]	*n* = 1301; international pragmatic RCT; nutritionally high-risk mechanically ventilated adults; 85 ICUs in 16 countries	Prescribed protein ≥ 2.2 versus ≤1.2 g/kg/day. Higher protein did not improve time to discharge alive or 60-day mortality and showed potential harm in AKI and greater organ dysfunction.	Does not support routine early high-dose protein and indicates the need for caution in AKI.
**Lee et al.** 2024 [35]	23 RCTs; *n* = 3303; meta-analysis and trial sequential analysis	Higher protein did not reduce overall mortality. Mortality was increased in the AKI subgroup, and serum urea was higher.	Confirms the absence of overall benefit and supports caution in patients with AKI.
**Bels et al.** **PRECISe** 2024 [12]	*n* = 935 double-blind multicentre RCT; 10 Belgian and Dutch ICUs	Isocaloric protein delivery of 2.0 versus 1.3 g/kg/day. Higher protein worsened health-related quality of life, did not improve functional outcomes, and increased gastrointestinal intolerance.	Routine higher protein provision from ICU admission does not improve recovery and may worsen patient-centred outcomes.
**Heuts et al., 2025** [28]	22 RCTs; *n* = 4164; Bayesian meta-analysis	Higher versus lower protein delivery showed a low probability of clinically meaningful benefit for mortality or other patient-centred outcomes.	Reinforces the absence of established benefit from routine higher early protein delivery.
**Schouteden et al.—PRECISe Bayesian analysis, 2025** [32]	*n* = 935; pre-planned Bayesian reanalysis of PRECISe	The probability of benefit from high protein for health-related quality of life was 0%, while the probability of benefit for 60-day mortality was low. Estimates of mortality harm varied according to the prior assumptions.	Reinforces the original PRECISe findings and indicates a low probability of benefit from routine high early protein provision.
**Summers et al.** **TARGET Protein** 2025 [33]	*n* = 3397 cluster-randomized crossover trial; eight Australian and New Zealand ICUs	Isocaloric enteral formulas containing 100 versus 63 g protein/L. Augmented protein did not improve hospital-free days or 90-day survival.	Extends the absence of demonstrated benefit from augmented protein delivery to a broad ICU population.
**Badpeyma et al., 2025** [27]	*n* = 56; double-blind, two-centre RCT	Protein targets of 2.2 versus 1.0 g/kg/day with equal energy targets. Higher protein reduced in-hospital mortality and loss of mid-arm circumference, but not 60-day mortality.	Provides a potentially beneficial signal, but the small sample limits generalizability.

Notes: Studies are presented chronologically. Protein doses represent prescribed or achieved intake, as specified. Clinical implications reflect the interpretation of the evidence within the phase-adapted framework of this review. Abbreviations: AKI, acute kidney injury; ICU, intensive care unit; PN, parenteral nutrition; RCT, randomized controlled trial. Abbreviations: GI: gastrointestinal; HR: hazard ratio; NNH: number needed to harm; QoL: quality of life; RR: relative risk; TSA: trial sequential analysis; TTDA: time-to-discharge-alive. Bracketed numbers refer to the reference list.

## Data Availability

No new data were created or analyzed in this study. Data sharing is not applicable to this article.

## Supplementary Materials

The following supporting information can be downloaded at: https://www.mdpi.com/article/10.3390/nu18172840/s1, Figure S1: Flow diagram of the literature identification, screening, eligibility assessment, and publication-selection process.

## Abbreviations

The following abbreviations are used in this manuscript:

AKI	Acute Kidney Injury
ASPEN	American Society for Parenteral and Enteral Nutrition
CANH	Clinically Assisted Nutrition and Hydration
EMAS	Early Mobilization and Nutrition Study
EPaNIC	Early Parenteral Nutrition in Adult Critically Ill Patients
ESPEN	European Society for Clinical Nutrition and Metabolism
GLIM	Global Leadership Initiative on Malnutrition
GRADE	Grading of Recommendations Assessment, Development and Evaluation
GRIP	Global Research Initiative on Post-ICU Nutrition
ICU	Intensive Care Unit
ICU-AW	Intensive Care Unit-Acquired Weakness
mNUTRIC	Modified Nutrition Risk in the Critically Ill score
NRS-2002	Nutrition Risk Screening 2002
PICS	Post-Intensive Care Syndrome
PN	Parenteral Nutrition
QoL	Quality of Life
RCT	Randomized Controlled Trial
TSA	Trial Sequential Analysis
TTDA	Time-to-Discharge Alive
VCO_2_	Carbon dioxide production
