# Supplementary material for: Phase-Specific Nutritional Therapy in Critical Illness: When Protein May Help and When It May Harm, from Acute Metabolic Adaptation to Post-ICU Recovery
Source: Nutrients. 2026 Aug 29;18(17):2840. doi: 10.3390/nu18172840 (PMC13567492; doi:10.3390/nu18172840)
Supplement: Supplementary file 1 [file nutrients-18-02840-s001.zip › nutrients-4499194-supplementary.pdf]

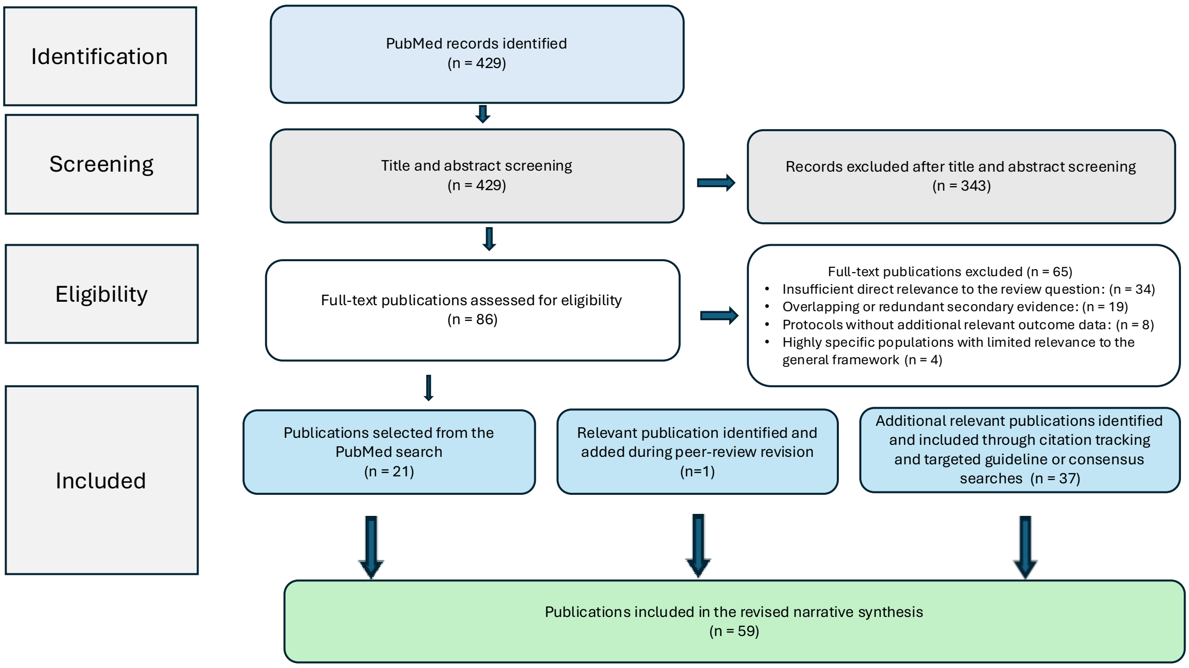

Figure S1. Flow diagram of the literature identification, screening, eligibility assessment, and publication-selection process. The figure is original and was created by the authors specifically for this review.

The final PubMed/MEDLINE search was conducted on 20 July 2026 and covered publications from January 2011 to June 2026. One additional relevant publication was identified and included during peer-review revision.
